# Retinal Diseases: The Next Frontier in Pharmacodelivery

**DOI:** 10.3390/pharmaceutics14050904

**Published:** 2022-04-21

**Authors:** Assaf Ben-Arzi, Rita Ehrlich, Ron Neumann

**Affiliations:** 1 Department of Ophthalmology, Rabin Medical Center, 39 Jabotinski St., Petah Tikva 4941492, Israel; assafbenarzi@gmail.com (A.B.-A.); ritaehrlich@gmail.com (R.E.); 2 Sackler School of Medicine, Tel Aviv University, P.O. Box 39040, Tel Aviv 6997801, Israel; 3 Department of Ophthalmology, Maccabi Sherutei Briut, Ramat Hasharon 4731001, Israel

**Keywords:** pharmacodelivery and drug delivery, global aging, age-related macular degeneration (AMD), diabetic retinopathy, anti-vascular endothelial growth factor (VEGF), implant, micro- and nanoparticles, micro- and nano-carriers, hydrogel, liposome, designed ankyrin repeat protein (DARP), Port delivery systems

## Abstract

The future continuous growth of the global older population augments the burden of retinal diseases worldwide. Retinal characteristics isolating and protecting the sensitive neuro-retina from the rest of the ocular tissues challenge drug delivery and promote research and development toward new horizons. In this review, we wish to describe the unmet medical needs, discuss the novel modes of delivery, and disclose to the reader a spectrum of older-to-novel drug delivery technologies, innovations, and the frontier of pharmacodelivery to the retina. Treating the main retinal diseases in the everlasting war against blindness and its associated morbidity has been growing steadily over the last two decades. Implants, new angiogenesis inhibitor agents, micro- and nano-carriers, and the anchored port delivery system are becoming new tools in this war. The revolution and evolution of new delivery methods might be just a few steps ahead, yet its assimilation in our daily clinical work may take time, due to medical, economical, and regulatory elements that need to be met in order to allow successful development and market utilization of new technologies. Therefore, further work is warranted, as detailed in this *Pharmaceutics* Special Issue.

## 1. The Burden of Retinal Disease in the Older Population

The impressive expansion of the global elderly population poses enormous challenges for human society in current times and also in the near future. The average human life expectancy has doubled in most developed countries in the last 200 years [1]. The global population is expected to reach 8.5 billion in 2030, 9.7 billion in 2050, and 10.9 billion in 2100. Today, approximately 9% of the global population is aged 65 and older, and the percentage is projected to reach nearly 12% in 2030, 16% in 2050, and 23% in 2100, with a much faster estimated rate of growth above the age of 80 years old. In 1990, there were 54 million people aged 80 and older in the world, and in 2019, there were nearly 143 million, almost three times more. Worldwide, the projected number of the elderly population above the age of 80 years in 2050 is expected to triple again to 426 million, and it may increase in the future to 881 million in 2100 [2] (Table 1).

The aging of the population is estimated to increase the health-related costs and burden on health systems. The major issue in the older population is the high prevalence of sight-threatening conditions: age-related macular degeneration (AMD), diabetic retinopathy, glaucoma, and cataract. AMD is the leading cause of blindness in elderly patients in developed countries. As for today, in 2020, approximately 196 million people worldwide are affected by AMD, with a global prevalence of 8.69%. According to latest predictions, the number of AMD patients will increase to 288 million people in 2040, as a consequence of the exponential increase in population aging [3]. Diabetic retinopathy (DR) was reported to be the only cause of blindness that showed a global increase in age-standardized prevalence analysis between 1990 and 2020. The reason for this increase is thought to be due to the greater longevity of diabetic patients. By 2040, predictions estimate more than 600 million people with diabetes worldwide, and, as a result, a rise is expected in vision impairment from DR [4].

The burden of retinal diseases in the elderly has a major impact on society and on the economy. Questionnaire research studies performed in Australian clinical settings have demonstrated that the cost of neovascular AMD is estimated to be between $5.15 billion and $5.75 billion per year; similar findings also come from the UK. The financial burden on the patient’s life and on society encompasses personal expenses, such as means for mobility, low-vision aids, and home modifications. In Canada, the estimations in 2007 of productivity loss, aids and home modifications, and the value of lost well-being were $4.4 billion, $305 million, and $11.7 billion, respectively. In addition, the indirect cost per patient was estimated to be $19,370 annually. These numbers underline the significant financial burden not only to the patient but also to the community and society. Furthermore, there is a significant influence on the patient’s quality of life, his/her surroundings, society involvement in activities, the ability to work, engagement in social activities, and maintaining the elderly social role in and outside their families, all leading to an immense need for support from social welfare systems, community services, and patients’ families. Moreover, there is a great burden on the patient’s environment, including the emotional, economic, and social aspects on care givers and families. Both the patient and his/her support group are prone to develop stress, anxiety, and depression; see their quality of life decline; and become unemployed [6,7,8,9]. 

The economic burden of macular diseases, including AMD and diabetic macular edema (DME), on patients, families, healthcare providers, and government systems may be classified as direct (medical and non-medical disease-related expenses) and indirect (informal care and productivity loss) costs. In a report by the AMD Alliance International in 2010, the estimated global direct cost of visual impairment due to AMD was expected to increase in the future decade from $255 billion to $294 billion in 2020, while indirect costs were expected to increase from $88 billion to $105 billion [10,11]. The global direct healthcare system costs and indirect costs were estimated to be approximately $2.77 trillion and $760 billion in 2020, respectively [11].

The potential support ratio is defined by the UN as the number of people of working age (25 to 64) for each person aged 65 years and older. This ratio today is above 5 and is even reaching 10 in developing countries, and the ratio is around 3 in developed countries. This ratio is expected to decline by 2050 to very low values, even below 2 in Europe, Northern America, and Southeastern Asia [2]. The expected increase in disease burden of age-related macular degeneration in the next 30 years raises the following objectives: prevention of disease and early identification of patients prior to the conversion to neovascular AMD while visual acuity (VA) is still preserved; avoiding undertreatment evidenced by real-world studies (e.g., AURA [12]) compared to clinical trials (e.g., VIEW [13] and CATT [14]) to ensure best possible outcomes; and reducing the treatment burden (e.g., frequent office visits, repeated imaging studies, and serial intravitreal injections) to improve both the patient’s quality of life and therapy experience [15]. Most important, we need effective therapies and means of delivery of therapies to the retina, while avoiding systemic adverse events and securing better vision.

## 2. The Physiological and Anatomical Challenges of Retinal Diseases Therapy

**Figure 1:**
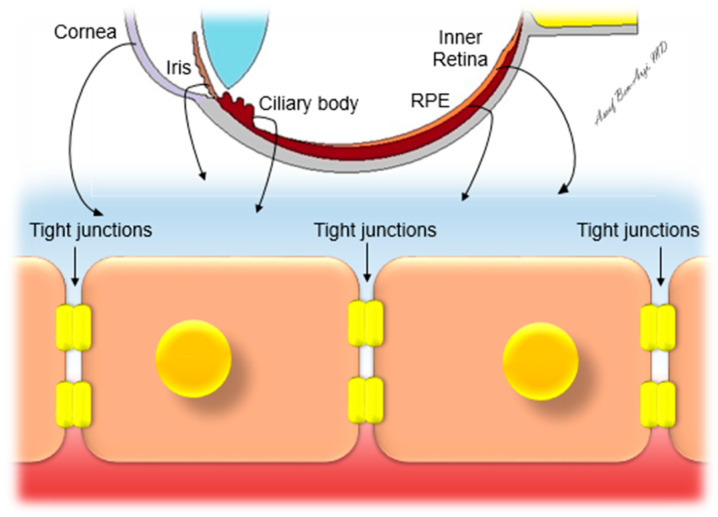
The various ocular barriers with tight junctions.

The eye is a unique and complex organ that is protected by tight defined barriers (Figure 1). These barriers sequester and isolate the eye from the rest of the body by providing a privileged environment that allows for the normal function of the neuronal visual cells and maintenance of ideal optical conditions. The blood–ocular barrier consists of two systems: the blood–aqueous barrier (BAB) and the blood–retinal barrier (BRB). Those two systems regulate internal ocular tissues and fluids, resulting in a unique biochemical environment that differs from the external systemic circulation. The metabolically active pumps serve as a drainage route for the “waste-products” of photo-transduction. The transcellular active transport mechanisms include ATP-dependent processes such as receptor-mediated vesicular transport, non-receptor-mediated pinocytosis, transporters, and pumps. Examples of the latter include the following: sodium pump (Na^+^, K^+^/ATPase), sodium–potassium–two chloride (Na^+^/K^+^/2Cl^−^) cotransporter, sodium–hydrogen exchanger, chloride–bicarbonate exchanger, and sodium–calcium exchanger [16]. Both systems are based on tight junctions (TJ, zonulae occludentes).

The BAB is a system dedicated to forming two functionally separate and unique environments anterior and posterior to the iris. The posterior chamber is essentially free of plasma protein, while the anterior chamber contains minute amounts of plasma protein. This system is composed of tight junctions connecting the posterior pigmented epithelial cells and of the one-way valve created by the apposition of the pupillary margin and the anterior lens capsule, combined with the continuous forward flow of aqueous humor through the pupil [17].

The BRB is composed of inner and outer components. The inner BRB tightly connects neighboring retinal endothelial cells and restricts permeability. The inner barrier is made of a basal lamina that is covered by the processes of astrocytes and Muller cells and is thought to be influenced by these adjacent cells. The outer BRB connects neighboring retinal pigment epithelial (RPE) cells. It is composed of a single cell layer which lies upon the Bruch membrane, separating the neural retina from the fenestrated choriocapillaries and, thus, regulating the access of nutrients from the blood to the photoreceptors and maintaining retinal adhesion. The TJ protein complexes polarize the cells, with distinct apical and basal membrane surfaces, restricting paracellular diffusion of blood-barrier compounds into the neuronal tissues. The pathologic changes of the TJs result in increased permeability in diabetic retinopathy (inner BRB primarily affected) and neovascular AMD (outer BRB primarily affected) [18].

The treatment of retinal diseases is challenged by these ocular barriers, which prevent effective intraocular absorption of pharmaceuticals given. Topical therapy (eye drops), while effective in the management of some anterior ocular diseases, does not reach therapeutic concentrations in the posterior segment, due to the difficulties of substances to cross the cornea, the distance of diffusion, and the direction of intraocular outflow that directs those molecules that penetrated the eyeball out of it. The other possibility of drug delivery is an invasive procedure by means of intravitreal injection of drugs or implants, with reasonable therapeutic concentrations to the posterior segment but a substantial risk of serious side effects, such as endophthalmitis, hemorrhage, and retinal detachment [19].

A future possibility for countering pathologic hyperpermeability due to damaged TJs is by targeting new molecules that control retinal angiogenesis and barrier genesis. One example is the Wnt signaling pathways, which maintain inner BRB integrity. Transient deactivation or gene suppression of the Wnt pathways loosens the inner BRB and, therefore, may promote drug delivery; thus, it may become a new potential drug delivery tactic [16,20].

## 3. The Pharmaceutical Challenges and Future of Retinal Drug Therapy

Today, we are witnessing a new and unique era in the treatment of sight-threatening retinal diseases. Intravitreal injections are the mainstay of delivery of drugs and have revolutionized patients’ prognosis dramatically compared to the photocoagulation approach. The medical management of intravitreal injections is frequently a prolonged, ongoing chronic treatment which obligates patients and physicians to follow a strict routine, with monthly injections, frequent clinic visits, and the need of repeated retinal imaging for monitoring and decision making. These circumstances augment the burden on the patient’s life and his/her surroundings, increase the workload on the physicians, and require supportive infrastructure by health systems. The delivery of pharmaceutical agents by invasive means is not without harm, and due to the clinical need of numerous and frequent intravitreal injections, it carries an increased risk of infection, bleeding, retinal detachment, and all the previously discussed life-changing medical burdens on patients and their support groups.

The current arsenal of therapy by intravitreal agents may be categorized into two large and main families of treatments, divided by their molecular target: anti-vascular endothelial growth factors (VEGF) and corticosteroids. Each treatment has a unique and different profile of advantages and disadvantages. The molecular features, together with their molecular weight, clearance rate, dissociation constant, and molar dose, influence therapeutic efficiency and duration and side effects. The latter include local effects on intraocular pressure, with long-term consequences and systemic cardiovascular effects leading to relative and absolute contraindications.

The novel molecules that are currently under research and development are aimed toward multiple therapeutic targets in the pro-angiogenesis inflammatory signaling pathways and consist of different VEGF isoforms and subtypes, placental growth factor (PIGF), integrin, platelet derived growth, tyrosine kinase, epidermal growth factor, and gene therapies. Future directions target new molecules in the cell signaling pathways that control retinal angiogenesis and barrier-genesis for promoting drug delivery to the retina [16,20]. Further innovations are better addressed in the rest of this paper. 

As previously discussed, the delivery of drugs is as critical as the drugs themselves—the current paper is addressing this critical aspect of retinal pharmaceutics. As of today the situation is not ideal and efforts are still in progress to augment the therapeutic features of present successful treatments by enhancing drug delivery settings. One strategy is the designed ankyrin repeat protein (DARPin) molecules, which are composed of small single-domain proteins that can selectively bind to multiple targets with greater durability and prolonged molecular half-life [21]. An example of this technology is Abicipar Pegol, which, unfortunately, was withdrawn, due to high intraocular inflammation rates. Another strategy is the fusion of a polyethylene glycol tail, which affects the half-life of a drug. Another approach is sustained-release technologies, for example, the Dexamethasone implant and Fluocinolone Acetonide implant that are already in use. Moreover, new port delivery systems include the office refillable sustained-delivery device of Ranibizumab, which delivers a constant concentration of the target molecule for more than 6 months. Several sustained-release platforms are in different stages of clinical development, delivering tyrosine kinase inhibitors. New strategies of gene therapy drug delivery systems are still in development and consist of adenoviral mediated therapy to insert a gene coding for an anti-VEGF antibody fragment (e.g., RGX-314 and ADVM-022) [15].

## 4. The Pathways and Modalities of Drug Delivery

The drug administration route of choice is influenced by several factors that are derived from the drug molecule, its toxicity, patient compliance, and adverse effects to the drug and the delivery system. As alluded to, reaching the posterior segment through the anatomical and physiological natural barriers of the anterior segment via eye drops is almost impossible. Oral and intravenous routes encounter the BRB and BAB, which demand increased doses, leading to a higher risk of systemic toxicity. Intravitreal injections have the advantage of a direct delivery, low dose with sufficient bioavailability, and minimal toxicity. However, invasive procedures carry considerable risks, including intraocular inflammation, infection, and elevated intraocular pressure (IOP). Therefore, other periocular routes are of great interest, including topical, subconjuctival, suprachoroidal, subretinal, and trans-scleral routes (Table 2).

**Figure 2:**
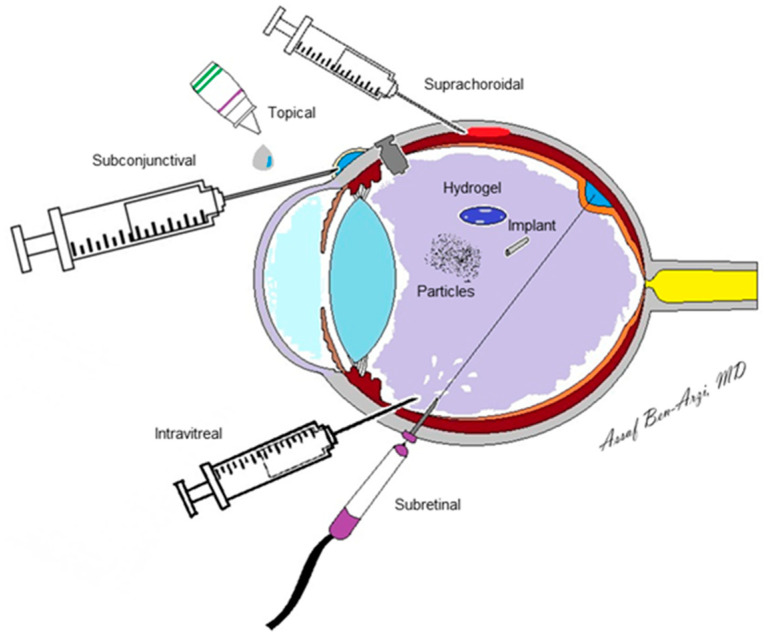
The various pathways of drug delivery to the posterior segment.

Retinal drug delivery encompasses a vast variety of technologies. A main objective of drug delivery systems is to extend the interval length between treatments. The significant advances in engineering have spawned technologies capable of achieving this goal: hydrogels, micro- and nanoparticles, and biodegradable implants, among others (Table 3). These technologies share the objectives to enhance intravitreal drugs’ half-lives, leading to better biocompatibility and reduced biological drug degradation. The methodologies through which drug technology is delivered to the eye also varies, i.e., improved eye drops, injections to the subconjunctival, suprachoroidal and subretinal space, trans-scleral port delivery systems, and intravitreal injections (as seen in Figure 2) [22].

In the past two decades, sophisticated drug delivery development has allowed us to slowly increase the interval length between intravitreal injections via improving drug substances and their release. Sometimes the drug material itself can result in shorter or longer timespan of the drug effect via different pharmacodynamic profiles. One example, Brolucizumab, the newest anti-VEGF drug approved by the FDA in 2019, is a single-chain antibody fragments with a small molecular weight (26 kDa) and higher tissue penetration, and it may be administered in some patients at a 12-week interval, as compared to the monthly injection of the previous anti-VEGF agents that are used today. In Aflibercept phase-three trials, VISTA and VIVID have demonstrated visual improvements through injection every 8 weeks. However, in real-life clinical settings, this regimen could be applied to some of the patients but not to all [23,24,25]. Brolucizumab demonstrated a superior anatomical outcomes compared to the latter in the HAWK and HARRIER trials; however, the adverse events of intraocular inflammation with obstructive retinal vasculitis are a cause of increased concern [15,21].

Extended drug bioavailability could be achieved by intravitreal implants. These implants may be classified as non-biodegradable (NB) and biodegradable. The first NB implantable intravitreal device, named Vitrasert^®^, was developed in 1992 for the treatment of cytomegalovirus retinitis with ganciclovir. The device is made of one restricted membrane limiting drug release (ethylene-vinyl acetate, EVA) and a frame of a permeable polymer regulating the diffusion of the drug into the vitreous (polyvinyl alcohol (PVA)). EVA is characterized by being a hydrophobic discontinued film that allows molecules to be dissolved in fluid and consequently diffused into the vitreous through the permeability of the PVA infrastructure set as inner and outer layers surrounding the EVA [26]. In 2005, the FDA approved the second NB implant, Retisert^®^, for the treatment of chronic noninfectious uveitis with fluocinolone acetonide (FA) conjugated to PVA with silicone layers surgically implanted and anchored to the sclera by sutures. While having successful outcomes in terms of visual acuity and reducing recurrences of uveitis in a period of three years, the main disadvantages were related to the prolonged intraocular presence of steroids: intraocular pressure increase leading to glaucomatous damage that did not respond to anti-glaucoma medication, necessitating surgical glaucoma intervention in about 40% of patients (e.g., trabeculectomy); and accelerated cataract formation was observed in 80% of patients requiring cataract extraction [26]. The smallest NB implant approved by the FDA in 2014, Illuvien^®^, is a cylindrical-shaped device made of PVA and silicone to deliver FA for the treatment of DME, injected intravitreally by a 25-gauge needle, creating a self-sealing wound. The greatest advantage consists of improvement of vision for up to 3 years; however, it also constitutes the main disadvantage—having an active steroid in the eye for such a long period of time without a good way, other than surgery, to halt the pharmaceutical action of the drug. General disadvantages of non-biodegradable implants include the following: dissociation; and all intraocular-surgery-related complications, such as hemorrhage, endophthalmitis, and risk of retinal detachment [26,27].

A representative biodegradable implant is Ozurdex^®^, a dexamethasone intravitreal implant composed of poly lactic-co-glycolic acid (PLGA), a synthetic aliphatic polyester predominantly biodegraded via non-enzymatic hydrolysis of the ester linkages under physiological conditions. The device is introduced via a 22-gauage self-sealing injection technique. The FDA approved it in 2011 for the treatment of DME with efficacy for up to 6 months. Reapplication or adjuvant therapy may be warranted for sustained functional and anatomical improvement [27,28]. Further indications were added along the years for the treatment of retinal vein occlusions and non-infectious uveitis affecting the posterior segment [29].

Micro- and nano-molecular-size carrier systems of drugs may also assist in crossing natural eye barriers; however, despite extensive research over the last decade and numerous developments, these technologies are yet to be approved. These carriers for ocular drug delivery include several options: liposomes, nanoparticles (NPs), micelles, and dendrimers, among others. Their advantages are in more effective tissue absorption and cellular uptake compared to larger particles; they can also be designed to have a controlled release mechanism and may be aimed at a specific target or destination. Liposomes are single- or double-layered lipid membrane structures with enhanced permeability and biocompatibility that enable them to encapsulate hydrophilic and hydrophobic drugs; NPs can extend the half-life of the carried drug, reduce frequency of application, and reduce toxicity and adverse events. NPs are colloidal carrier systems in the nano-metric dimension, generally around 50–500 nm, with the ability to actively or passively transport; they are designed to adapt to the ionic ambient of the vitreous collagen and glycosaminoglycan matrix. Micelles are nano-sized amphiphilic core–shell (hydrophobic core and hydrophilic shell) carriers with polymeric surfactants. Dendrimers are branched polymeric three-dimensional star-shaped nano-carriers. The most investigated dendrimers for drug and gene delivery are polyamidoamine (PAMAM), which are not biodegradable and still require further investigations regarding long-term safety and cytotoxicity for ocular use. An example for a microparticle drug delivery is the microcrystals depot of triamcinolone acetonide (TA) commercialized as Kenalog^®^ and Triesence^®^, with controlled release and long-lasting therapeutic effects, as well as a half-life time of up to 120 days in rabbits [30,31]. Another example of carrier systems is the use of Sunitinib malate, which is approved as a treatment for renal cell carcinoma. Sunitinib is a tyrosine kinase inhibitor targeting both VEGF-A and platelet-derived growth factor (PDGF). Sunitinib for the treatment of retinal vascular disease is a promising agent, and it is still in clinical research. Several drug delivery technologies were developed combining Sunitinib, including the following: solid lipid nanoparticles studied in rabbits and microparticles demonstrated in a phase-two clinical trial to have a steady therapeutic effect in the choroid and retina for more than 6 months; however, this advanced technology has yet to be approved for clinical use [22,32,33,34,35,36,37,38,39].

Another type of a potential carrier is the hydrogel formulation. As of today, it is used in clinical research utilizing soft contact lenses to deliver drugs to the anterior segment. This technology is still being researched. Hydrogels are well-defined structures that are composed of a network of hydrophilic monomers with crosslinked bonds. The designated components and variable crosslinked bonds that respond to physical and biological stimuli from the surrounding environments enable and control density, porosity, and mechanical strength metabolism, and, thereby, they control drug release. These environmental stimuli have a range of temperature differences, photo-stimulation levels, pH levels, and ionic strength levels. While the anterior segment applications of the hydrogel are present with soft contact lenses and foldable intraocular lenses, the progress of the development of posterior segment applications is slow and challenging. Some of these challenges are sterilization without changing structure; removal of toxic agents; limited shelf-life; linking to hydrophobic and macromolecular drugs; intravitreal delivery, leading to additional in situ crosslinking inside the vitreous body; and related toxic side effects. The long-term effects are unknown, and the high variability in degradation rate and hydrogel swelling could increase IOP and induce the occlusion of retinal vasculature. Research is still ongoing, combinations of technologies of hydrogels together with micro and nano-particles may also enhance drug delivery. Experiments with an in vitro and a nonhuman primate model of a biodegradable thermo-responsive microsphere hydrogel carrying Aflibercept demonstrated efficacy for up to 6 months of controlled release drug post-injection [40,41,42,43,44].

The port delivery system, named Susvimo, is a nondegradable, refillable implant that is surgically placed through the sclera in the pars plana, with passive diffusion of drug molecules from the port to the vitreous cavity, and sustained and controlled release achieved by the porous metal element. A phase-two clinical trial demonstrated a median time of 15 months for an initial refill of Ranibizumab, with visual outcomes similar between patients administered the Ranibizumab-loaded port 100 mg/mL and monthly intravitreal injections after 9 months. Archway, a phase-three study was conducted and concluded for the treatment of AMD and supported the recently approval from the FDA. Phase-three clinical trials in progress are held for DME (PAGODA) and DR (PAVILION) [45].

Eye drops as topical administration are an obvious objective for drug delivery, thanks to the great advantages of being a non-invasive self-care route of treatment. However, drug bioavailability and efficiency to the posterior segment are still a major challenge. Research and development are still ongoing, with controversial outcomes that make them not yet mature enough for medical use [46,47]. A recent publication of a nanomicelles capable of delivering Aflibercept topically to the posterior segment demonstrated choroidal neovascularization regression [48]. In current clinical use, Difluprednate (Durezol^®^), for example, is a potent steroid emulsion for topical administration. In a study conducted on rabbits, it was detectable in the posterior segment. Furthermore, it demonstrated a similar anatomical outcome in persistent DME compared to subtenon TA in one research and was even more effective than betamethasone in visual acuity outcomes in another small study [49,50,51]. Difluprednate significantly increased intraocular pressure more than other steroids and therefore is limited in clinical use [52,53,54]. Nepafenac (Nevanac^®^) is a topical NSAID suspension. There are some reports of penetration to the posterior segment in animal models. It is used to prevent and treat pseudophakic cystoid macular edema and has a low profile of adverse events compared to steroids [55,56,57]. 

Other invasive, but not via the vitreous, routes include subconjunctival, subtenon, and suprachoroidal routes. The subconjunctival space is rich in blood and lymphatic vessels, increasing the systemic absorption of small molecular drugs and, thus, possessing limited efficiency regarding the posterior segment [58]. The subtenon may serve as a route for absorption to the posterior segment; however, due to the choroidal circulation flow, the efficiency is reduced. The suprachoroidal route may enable a targeted controlled release route with drug accumulation; nevertheless, it has a substantial risk of choroidal detachment and hemorrhage [59]. New developments and research are still in progress [60,61,62], with some phase-three studies conducted. The results were mixed, with some studies reaching their primary end points and some not. As of today, regulatory approval has not been granted yet.

The subretinal route targets the potential space between the RPE and photoreceptors and is achieved via microsurgery, with the great advantage of direct effect to the retina [63]. A recent publication reported the pioneering surgical experience of robot-assisted subretinal drug delivery of tPA in humans under local anesthesia [64]. Another example is the DNA-carried viral vectors that were injected into the subretinal space for treating inherited retinal diseases such as retinitis pigmentosa and Leber’s congenital amaurosis [65].

Moreover, gene therapy technology is being researched. Among others, transforming retinal cells to produce anti-VEGF drugs, thus reducing the injection-burden of current therapy. For example, the RGX-314 is being developed to transform cells so that they can produce anti-VEGF antibodies Fab fragments through genomic constructs injected to the subretinal and suprachoroidal space. In phase 1/2, the burden of anti-VEGF treatment was reduced, with up to 85% fewer injections after the application of the gene therapy. Adverse effects were seen; among them was inflammation in 36% of patients. Another example is ADVM-022 encoding Aflibercept genetically for treating neovascular AMD, carried by adeno-virus vectors injected directly to the vitreous. In this case, as well, inflammation has been developed in one case and was diagnosed as a recurrent moderate uveitis. A substantial decrease in anti-VEGF injections was achieved thereafter [15,66,67]. A different strategy, small interfering ribonucleic acid (siRNA) molecules, are double-stranded RNA that can inactivate a messenger RNA (mRNA) target, leading to gene silencing. So far, siRNA drugs have been studied in phase 1/2 for targeting AMD, DME, and congenital retinal disorders; however, adverse events were reported, thus limiting the utilization in clinics today [68].

A minimally invasive drug delivery route which requires further research for understanding its clinical implication is the trans-scleral iontophoresis, a method which manipulates low electric current applied to the sclera to enhance the transport of molecules to the posterior segment with passive diffusion. Studies have been performed on experimental uveitis models for inflammation suppression, and other studies have demonstrated efficiency on choroidal neovascularization models with anti-VEGF; however, further research is necessary [69,70].

## 5. Conclusions

We are in an exciting time with many developments in pharmacology and technology that have the potential to revolutionize our treatments to prevent blindness. As it seems, in several studies, the combining technologies together is a key for success with promising results. However, challenges remain, and still much work is needed before actual real-world, day-to-day therapy results will be seen with these revolutionary drug delivery strategies. Further research on and development of devices and novel molecules in different signaling pathways for better retinal drug delivery strategies are still warranted. We believe that the near future holds promising advancements of technologies in the ophthalmologic world for the management of posterior segment diseases.

## Figures and Tables

**Table 1:** Global assessments of human population and major retinal disease. Data adapted from the following: [1,2,3,4,5].

Human Population in Numbers				
**2020**	**2030**		**2050**	**2100**
7.7 billion	8.5 billion		9.7 billion	10.9 billion

**Percentage of People above the Age of 65 Years**				
**2020**	**2030**		**2050**	**2100**
9%	12%		16%	23%

**The Number of People above the Age of 80 Years**				
**1990**	**2020**		**2050**	**2100**
54 million	143 million		426 million	881 million

**AMD Patients in Numbers**			**Diabetic Retinopathy Patients in Numbers**	
**2020**	**2040**		**2020**	**2045**
196 million	288 million		103 million	160 million

**Table 2:** Experience of drug delivery via various technologies.

Drug Delivery Pathway	Description	Example
Topical	Non-invasive easy to apply treatment. Usually drops or ointment applied to the surface of the eye.	Nepafenac
Subconjunctival	Injection targeted to the potential space between the conjunctiva and Tenon layers.	Triamcinolone acetonide
Subtenon	Injection targeted to the potential space between the Tenon and the sclera.	Triamcinolone acetonide
Suprachoroidal	Injection targeted to the potential space between the sclera and the choroid.	Gene therapy, Triamcinolone acetonide
Subretinal	Injection targeted to the potential space between the choroid and the retina. Could be applied externally or internally intraoperatively through the vitreous via pars plana vitrectomy.	Gene therapy, tissue plasminogen activator (tPA)
Trans-scleral		
Port delivery system	Refillable implant device anchored to the sclera, allowing for diffusion of drug molecules to the vitreous.	Ranibizumab port delivery system (Susvimo^®^)
Iontophoresis	Manipulation of low electric current applied to the sclera for passive diffusion of molecules intraocularly.	In clinical research
Intravitreal		
Injections	Injection to the vitreous cavity of drugs.	Anti-VEGF, steroids
Implants	Injection to the vitreous cavity of implants carrying drugs with a sustained release mechanism.	Dexamethasone biodegradable implant (Ozurdex^®^)

**Table 3:** Leading drug delivery modalities.

Drug Delivery Molecular Modalities	Size\Mass	Structure\Formulation	Status
Drug dose escalation			
e.g., Brolucizumab, Beovu^®^	26 kDa	Single chain antibody fragment	In clinical use
Sustained release intravitreal implants			
Biodegradable			
e.g., dexamethasone intravitreal implant, Ozurdex^®^	0.46 × 6 mm	Rod shaped, PLGA	In clinical use
Non-biodegradable			
e.g., fluocinolone acetonide intravitreal implant, Retisert^®^	2 × 5 mm	Pellet, PVA and silicone laminate	In clinical use
Sustained release carriers			
Designed ankyrin repeat protein (DARP)			
e.g., Abicipar pegol	34 kDa	Antibody mimetic proteins packed in DARP technology	Withdrawn in clinical trials
Liposome	25 nm–2.5 μm	Vesicles of lipid layers	In clinical research
Micro particles			
e.g., Triamcinolone acetonide, Triesence^®^, Kenalog^®^	1–100 μm	Microcrystals	In clinical use
Nano particles	1–1000 nm	Solid lipid, coated biodegradable polymers	In clinical research
Hydrogel	1–1000 nm	Network wide variety of hydrophilic monomers connected with crosslinked bonds	In clinical research
Port delivery systems			
e.g., Ranibizumab refillable injection device, Susvimo^®^	4.6 × 8.4 mm	Trans-scleral device, self-sealing septum, body reservoir and titanium release control element.	FDA approved
Adeno-associated-virus vector gene therapy			
e.g., small interfering RNA (siRNA)	20 nm	Icosahedral nucleocapsid containing the gene therapy sequence	In clinical research
e.g., voretigene neparvovec-rzyl, Luxturna^®^			FDA approved

## Data Availability

Not applicable.
